# The impacts of corrected left ventricular ejection time combined with dobutamine on hepatocellular carcinoma patients

**DOI:** 10.1038/s41598-022-24907-x

**Published:** 2022-12-07

**Authors:** Yu Jian, Ji Xiaochen, Sun Zongjian, Nie Yu, Shan Shiqiang

**Affiliations:** Department of Anesthesiology, Cangzhou Central Hospital, Cangzhou, 061000 Hebei China

**Keywords:** Diseases, Health care, Medical research

## Abstract

To evaluate the clinical effect of corrected left ventricular ejection time (LVETc) combined with dobutamine on the intraoperative management of patients undergoing hepatectomy for hepatocellular carcinoma. Sixty-eight patients with elective proposed pancreaticoduodenectomy, aged 61–78 years, body mass index 19–26 kg/m^2^, and ASA classification II or III, were divided into two groups (n = 34) using the random number table method: the esophageal ultrasound group (S group) and the esophageal ultrasound combined with dobutamine group (D group). In both groups, an esophageal ultrasound probe was placed after induction of anesthesia, and the left ventricular ejection time (LVET) and stroke volume (SV) were measured via a long-axis section of gastric fundus to guide fluid infusion. Nitroglycerin or a combination of dobutamine and nitroglycerine were pumped intravenously from the beginning of surgery to the completion of hemostasis after partial hepatectomy, in groups S or D, respectively. Central Venous Pressure (CVP), heart rate HR, and mean arterial pressure MAP were recorded at entry (T_0_), immediately after induction (T_1_), at the beginning of the operation (T_2_), during hilar occlusion (T_3_), after partial hepatectomy (T_4_), and after the operation (T_5_). SV and LVETc were recorded between T_1_ and T_5_. At T_0_ and T_5_, blood samples from radial artery and central vein were taken to determine the concentration of blood lactic acid, and the oxygen supply index (DO_2_I) and oxygen uptake rate (O_2_ERe) were calculated by blood gas analysis. The operation time, hilar occlusion time, intraoperative urine volume, intraoperative crystalloid and colloid infusion, intraoperative blood loss and blood transfusion, and the occurrence of cardiac gas emboli during the operation were also recorded. Adverse events of cardiovascular, pulmonary, and renal function during and after operation were registered. Sixty-four patients were included in the final analysis. Compared with group S, group D had lower CVP values at T_2_–T_3_ and higher SV values at T_2_–T_5_, reduced intraoperative blood loss, significantly increased intraoperative urine output, a smaller total dose of nitroglycerin use, and lower incidences of intraoperative hypotension and cardiac gas emboli (*P* < 0.05). Esophageal ultrasound detection of LVETc combined with dobutamine ensures hemodynamic stability in patients undergoing partial hepatectomy while reducing the incidence of intraoperative hypotension and air embolism.

## Introduction

Primary liver cancer is one of the most frequently occurring malignant tumors in China, and is associated with high morbidity and mortality rates^1^. Partial hepatectomy is an effective treatment for patients with liver cancer because it can prevent future recurrence and ensure that enough functional parenchyma remains. In addition to current literature, the guidelines for perioperative care for liver surgery recommend the intraoperative use of the controlled low central venous pressure (CLCVP) technique due to its ability to reduce hepatic venous stasis, intraoperative bleeding, and perioperative time during liver resection^2–4^. Esophageal ultrasound is a non-invasive and safe imaging modality that allows real-time monitoring of cardiac function and hemodynamic changes, but also the timely adjustment of blood volume. Furthermore, this procedure can also detect liver lesions and verify the diagnosis of liver metastasis. It has been confirmed that the corrected left ventricular ejection time (LVETc) correlates with left heart preload and instantly reflects left heart systolic function^5^, thus guiding intraoperative volume management, maintaining appropriate volume status in patients, and reducing postoperative cardiovascular adverse events. Ryu et al*.*^6^ found that the use of milrinone, an inotropic drug indicated for cardiac support in patients with heart failure, in liver surgery resulted in better reduction of central venous pressure and maintenance of intraoperative hemodynamic stability. Therefore, this study used esophageal ultrasound to detect LVETc and guide the volume status of patients undergoing partial hepatectomy. Furthermore, the effect on the CLCVP technique was explored by enhancing myocardial contractility with dobutamine to provide a clinical reference.

## Methods

### Inclusion/exclusion criteria

Sixty-eight patients undergoing elective laparoscopic partial hepatectomy (resection of ≥ 2 liver lobes) were included in this study. Inclusion criteria were as follows: aged between 61 and 78 years, body mass index of 19–26 kg/m^2^, ASA classification of II or III, and a Child–Pugh score of 3–7. Furthermore, patients were included if they had no preoperative pulmonary infection, no severe hepatic or renal insufficiency, no history of severe cardiovascular or cerebrovascular disease or hypertension, no coagulation dysfunction; no anesthesia, no history of drug allergy, and no history of esophageal or gastric disease. In contrast, patients were excluded from this study if they had: intraoperative bleeding > 800 ml; systolic blood pressure (SBP) < 90 mmHg or mean arterial pressure (MAP) < 65 mmhg; no response to vasoactive drugs for more than 10 min.

Using the random number table method, patients were divided into two groups (n = 34): patients who only underwent esophageal ultrasound (group S) and patients who underwent esophageal ultrasound combined with dobutamine (group D).

### Surgical procedures

Following patient admission to the surgical room and induction and maintenance of anesthesia, MAP, Pulmonary oximetry (SpO_2_), HR, Electrocardiogram (ECG), End-tidal Carbon Dioxide Tension (PETCO_2_), Bispectral index (BIS), and nasopharyngeal temperature were monitored. Furthermore, peripheral venous access was established, a left radial artery puncture tube was placed under local anesthesia, and a transducer was connected to monitor arterial pressure in real time. Under local anesthesia, a central venipuncture was performed and cannulated through the right internal jugular vein, fluids were infused, and central venous pressure (CVP) was monitored. All patients were intubated under general anesthesia, and they received etomidate 0.3 mg/kg, cis-atracurium 0.2 mg/kg, sufentanil 0.3–0.5 µg/kg for intravenous induction. Consequently, patients were connected to an anesthesia machine (Dreager, Germany) for mechanical ventilation after tracheal intubation and respiratory parameters were adjusted as follows: inhalation oxygen concentration: 2 L/min; tidal volume: 8–10 ml/kg; ventilation frequency: 12 times/min; inspiration-expiration ratio: 1:2; ventilation frequency: 12 times/min; and inspiration-to-expiration ratio: 1:2. Anesthesia was maintained with intravenous infusion of propofol 4–6 mg kg^−1^ h^−1^, target-controlled infusion of remifentanil (Minto model, plasma target concentration 2–3 ng/ml), intermittent intravenous administration of cis-atracurium 0.1 mg/kg, maintenance of BIS between 40 and 60, P_ET_CO_2_ at 35–45 mmHg, while maintaining nasopharyngeal temperature using a warming blanket and fluid warmer at 36.5 °C or higher. An ultrasound probe was placed transesophageally to obtain multiple cardiac views for monitoring SV, LVETc, and the occurrence of air bubbles in the cardiac chambers. The intraoperative laparoscopic pneumoperitoneum pressure was ≤ 12 mmHg.

### Measurements and records

CVP, HR, and MAP were recorded at entry (T0), immediately after induction (T1), at the beginning of the operation (T2), during hilar occlusion (T3), after partial hepatectomy (T4), and after the operation (T5), and SV and LVETc were recorded at T1–T5. At T0 and T5, blood samples from radial artery and central vein were collected to determine the concentration of blood lactic acid, and the oxygen supply index (DO2I) and oxygen uptake rate (O2ERe) were calculated by blood gas analysis. The bleeding volume was assessed according to the volume of blood aspirated in the suction device and the weight of the gauze blood pad, where the volume of blood aspirated by gauze (ml) was defined as:

$${\text{weight of gauze after aspiration }}\left( {\text{g}} \right) - {\text{weight of gauze before aspiration }}\left( {\text{g}} \right)/1.054 \; \left( {{\text{average density of blood}},{\text{ g}}/{\text{ml}}} \right).$$

We also recorded various parameters, such as the operation time, hepatic portal block time, intraoperative urine volume, intraoperative crystalloid and colloid infusion volume, and intraoperative vasoactive drug use. Furthermore, the occurrence of intraoperative and postoperative cardiovascular adverse events (hypertension, hypotension, heart failure and arrhythmia, etc.), postoperative pulmonary complications (pneumonia, pulmonary edema, etc.), and renal insufficiency (postoperative oliguria, anuria, etc.) were documented. Intraoperative cardiac gas embolism was recorded, and cardiac CO_2_ gas embolism was graded into five classes based on the proportion of bubbles detected: class 0, no bubbles seen in multiple views; class 1, non-contiguous visible single bubbles (< 10/section); class 2, non-contiguous visible clusters of bubbles (< 10/section); class 3, continuous visible clusters of bubbles (> 20/section); and class 4, continuous clusters of bubbles (> 20/section) in the right heart system^7^.

### Medications

In both groups, patients received 5 ml/kg of sodium lactate Ringer's solution for compensatory volume expansion 30 min before induction of anesthesia and 5 ml/kg/h of sodium lactate Ringer's solution as a background infusion after tracheal intubation. The esophageal ultrasound probe was placed approximately 45 cm from the incisors after induction of anesthesia, and the probe angle and visualization depth were adjusted. LVET and SV were measured by the Doppler method a long-axis section of gastric fundus, and LEVTc was calculated using the following equation: (LEVTc = LEVT × HR/60). Based on LVETc and SV results, fluid infusion was then guided accordingly. The patient's blood volume was insufficient when LVETc < 0.35 s, and 3 ml/kg succinyl gelatin injection was then provided within 10 min. In contrast, if SV remained unchanged or increased, while LVETc < 0.35 s, fluid therapy was continued at the same rate. If LVETc > 0.35 s, but SV increased more than 10% (using the basal value after induction of anesthesia as the reference), fluid therapy was continued at the same rate. If LVETc > 0.4 s and SV increased less than 10%, fluid therapy was discontinued until LVETc decreased to more than 10%, and the above rehydration process was repeated to control LEVTc between 0.35 and 0.40 s.

Group S: A nitroglycerin dose of 0.3ug/kg/min was administered for intravenous pumping from the beginning of surgery to the completion of hemostasis after partial hepatectomy, and the pumping rate was gradually increased according to CVP to maintain CVP ≤ 5cmH_2_O, MAP ≥ 65 mmHg, and HR < 100 times/min. However, if MAP < 65 mmHg, norepinephrine (0.01–0.1 µg/kg/min) was continuously pumped to ensure that MAP was ≥ 65 mmHg.

Group D: Continuous pumping of 3ug/kg/min dobutamine plus 0.3ug/kg/min of nitroglycerin were administered from the beginning of surgery to the completion of hemostasis after partial hepatectomy. The remaining parameters were the same as in group S.

Intravenous atropine 0.2–0.3 p.p. and intravenous esmolol 10–20 p.p. were administered when sinus bradycardia (HR < 50 beats/min) and sinus tachycardia (HR > 100 beats/min) occurred, respectively. When the urine volume was less than 0.5 ml/kg/h, 10 mg of furosemide was injected intravenously. Concentrated red blood cells were input according to the patient's bleeding volume and hemoglobin concentration (the hemoglobin concentration needed to be more than 8 g/L in both groups after surgery).

### Statistical analysis

Statistical analyses were performed with the SPSS v.19.0 statistical software. Normally distributed measurement data were expressed as mean ± standard deviation ($\overline{x}$ ± s), and the independent sample t-test was used for comparison between groups. Repeated measurement data ANOVA was used to compare each index among different time points within groups, and the χ^2^ test was used for comparison of count data. Differences were considered statistically significant at *P* < 0.05.

### Ethics declarations

This study was approved by the Medical Ethics Committee of the Cangzhou Central Hospital (2019-056-01), and informed consent was signed with the family. All methods were performed in accordance with relevant guidelines and regulations.

## Results

Sixty-four patients were included in the final analysis. Four patients were excluded, three patients from group S with intraoperative bleeding greater than 800 ml, and one patient in group D who was insensitive to antihypertensive drugs and whose intraoperative blood pressure was not maintained. There were no statistically significant differences between the general conditions of the two groups for each index (*P* > 0.05), as shown in Table 1.

**Table 1:** Comparison of the general conditions of patients in the two groups ($\overline{x}$ ± s).

Group	n (cases)	Sex ratio (man/woman)	Age (year)	BMI)	Surgery time (min)	Hepatic portal occlusion time (min)	Left ventricle ejection fraction pre-surgery (%)	Left ventricle end-systolic volume pre-surgery (ml)
Group S	31	17/16	69.2 ± 6.8	20.8 ± 1.6	267.4 ± 73.1	82.4 ± 17.4	61.5 ± 8.6	35.5 ± 12.7
Group D	33	20/11	68.7 ± 5.9	22.9 ± 2.5	281.6 ± 69.5	87.6 ± 15.2	62.7 ± 9.2	37.5 ± 11.3

As shown in Table 2, MAP values at T_1_ –T_4_, HR values at T_1_, and CVP values in group S and group D at T_3_–T_4_ and T_2_–T_4_ , respectively, were reduced compared with T_0_ (*P* < 0.05). CVP values in group D at T_3_–T_4_ were reduced compared with group S (*P* < 0.05), and SV and LVETc were increased at T_2_–T_5_ in both groups compared with T_1_ (*P* < 0.05). Finally, SV values in group D were increased at T_2_–T_5_ compared with group S (*P* < 0.05).

**Table 2:** Comparison of hemodynamic and tissue perfusion indices between the two groups of patients ($\overline{x}$ ± s).

Indicators	Group	T_0_	T_1_	T_2_	T_3_	T_4_	T_5_
MAP (mmHg)	Group S	92.4 ± 14.2	81.4 ± 15.6^a^	86.2 ± 13.2^a^	82.3 ± 12.7^a^	80.5 ± 16.2^a^	90.0 ± 11.2
	Group D	99.8 ± 13.4	82.5 ± 11.2^a^	84.7 ± 12.5^a^	86.6 ± 11.5^a^	81.7 ± 14.7^a^	92.8 ± 13.4
HR (time/min)	Group S	72.2 ± 11.6	97.3 ± 10.1^a^	72.4 ± 13.2	72.3 ± 10.3	74.3 ± 9.9	71.1 ± 8.5
	Group D	76.3 ± 11.1	94.5 ± 10.7^a^	77.6 ± 10.8	74.3 ± 11.3	72.7 ± 10.5	73.7 ± 8.3
CVP (cmH_2_O)	Group S	9.1 ± 1.9	9.0 ± 2.0	7.8 ± 2.1	5.8 ± 1.5^a^	5.1 ± 1.7^a^	9.4 ± 1.8
	Group D	9.2 ± 1.8	9.1 ± 1.8	6.5 ± 2.0^ac^	3.7 ± 1.7^ac^	4.4 ± 1.6^a^	8.9 ± 1.9
SV (ml/time)	Group S		49.2 ± 8.3	57.1 ± 8.2^b^	58.6 ± 6.2^b^	61.4 ± 9.2^b^	65.6 ± 7.3^b^
	Group D		47.4 ± 9.2	64.7 ± 9.3^bc^	67.4 ± 6.3^bc^	72.7 ± 8.1^bc^	75.4 ± 7.2^bc^
LVETc (ms)	Group S		291 ± 12	334 ± 15^b^	355 ± 14^b^	359 ± 17^b^	367 ± 16^b^
	Group D		288 ± 14	357 ± 20^b^	364 ± 13^b^	367 ± 12^b^	378 ± 15^b^
aLac (mmol/L)	Group S	0.9 ± 0.3					1.3 ± 0.9
	Group D	0.8 ± 0.3					1.2 ± 0.7
DO_2_I (ml/min/m^2^)	Group S	571.3 ± 67.5					582.7 ± 37.6
	Group D	552.6 ± 64.7					547.7 ± 51.7
O_2_ERe (%)	Group S	24.4 ± 7.3					21.4 ± 6.1
	Group D	24.0 ± 6.5					24.8 ± 7.5

Compared with T_0_, ^a^*P* < 0.05; compared with T_1_, ^b^*P* < 0.05; compared with group S, ^c^*P* < 0.05.

**Figure 1:**
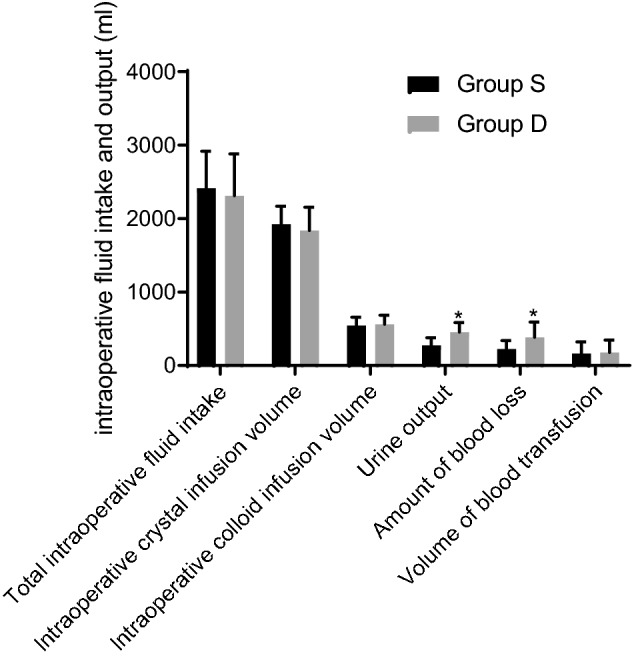
Intraoperative volume input and output of the two patient groups. Compared with the S group, the intraoperative blood loss in the D group was reduced, while the intraoperative urine volume was significantly increased. In addition, the difference was statistically significant (compared with the S group, *P < 0.05). However, there was no significant difference in the total fluid input, crystalloid infusion volume, colloid infusion volume and blood transfusion volume between the two groups (*P* > 0.05).

Compared with group S, intraoperative blood loss was reduced and intraoperative urine volume was significantly increased in group D, and the difference was statistically significant (*P* < 0.05). There were no statistically significant differences in total fluid intake, crystal infusion, colloid infusion, and blood transfusion volume between the two groups (P > 0.05), as shown in Table 3 and Fig. 1.

**Table 3:** Comparison of intraoperative fluid intake and output between the two groups of patients ($\overline{x}$ ± s).

Group	Total intraoperative fluid intake (ml)	Intraoperative crystal infusion volume (ml)	Intraoperative colloid infusion volume (ml)	Urine output (ml)	Amount of blood loss (ml)	Volume of blood transfusion (ml)
Group S	2413.6 ± 503.3	1924.2 ± 243.3	543.4 ± 116.0	274.2 ± 105.3	224.7 ± 117.2	164.5 ± 157.2
Group D	2312.4 ± 568.5	1837.4 ± 316.7	562.1 ± 122.4	453.5 ± 132.4^a^	386.2 ± 205.4^a^	178.2 ± 168.5

Compared with the group S, ^a^*P* < 0.05.

**Figure 2:**
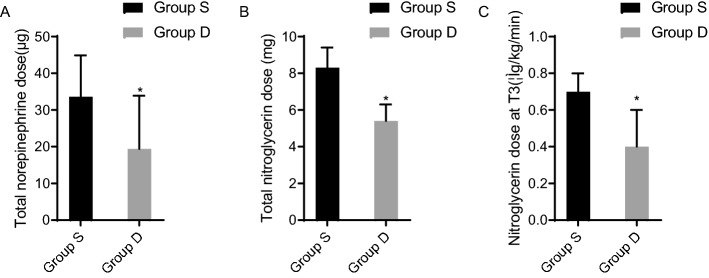
The use of vasoactive drugs in the two patient groups. Compared with the D group, the total dose of norepinephrine and nitroglycerin used in the S group was larger, and the difference was statistically significant (*P < 0.05). The dose of nitroglycerin used in group S at T3 was higher than that in group D, and the difference was statistically significant (*P* < 0.05). The dose of nitroglycerin used in group S at T3 was higher than that in group D, and the difference was statistically significant (*P* < 0.05).

As shown in Table 4, the total intraoperative doses of norepinephrine and nitroglycerin used were greater in patients in group S compared with group D, and the difference was statistically significant (*P* > 0.05). The dose of nitroglycerin used at T_3_ was greater in group S than in group D, and the difference was statistically significant (*P* < 0.05, Fig. 2).

**Table 4:** Comparison of intraoperative vasoactive drug use between the two groups ($\overline{x}$ ± s).

Group	Total norepinephrine dose (μg)	Total nitroglycerin dose (mg)	Nitroglycerin dose at T_3_ (μg/kg/min)
Group S	33.6 ± 11.3	8.3 ± 1.1	0.7 ± 0.1
Group D	19.4 ± 14.5^a^	5.4 ± 0.9^a^	0.4 ± 0.2^a^

Compared with the group S, ^a^*P* < 0.05.

The incidence of intraoperative hypotension was lower among patients in Group D than Group S (11% in Group S and 6% in Group D, respectively; *P* < 0.05). Furthermore, there was a statistically significant difference in the incidence of intraoperative cardiac gas emboli between the two groups (22.6% *vs*. 15.2% of grade 1 bubbles and 19.4% *vs*. 12.1% of grade 2 bubbles in Groups S and D, respectively; *P* < 0.05). There were no postoperative complications of pulmonary or cardiovascular adverse events or renal insufficiency in any of the patients in both groups, and the postoperative survival rate was 100% in both groups.

## Discussion

The key to a successful liver surgery is to control intraoperative bleeding while removing the patient's liver with precision, thus maximizing the preservation of normal liver tissue. With hepatic portal block during liver surgery, intraoperative hepatic section bleeding is mainly due to hepatic venous reflux bleeding, while hepatic venous pressure is directly related to CVP^8^. CLCVP is an anesthetic technique that controls central venous pressure at 0–5 cmH_2_O and SBP at ≥ 90 mmHg^9,10^. A decrease in central venous pressure will reduce the pressure of the inferior vena cava and hepatic vein, minimizing blood leakage from the liver section after hepatic portal block during liver surgery.

According to Guyton's theory, the venous return curve and cardiac function curve are depicted together, with CVP being their intersection point. Therefore, CVP can be effectively reduced by enhancing cardiac function. Dubin^11^ showed that 3ug/kg/min is the appropriate dose of dobutamine for increasing cardiac output; hence, this dose was used in our study. Our findings demonstrate that the total amounts of nitroglycerin and norepinephrine used intraoperatively and the dose of nitroglycerin during hepatic portal block were lower in Group D than in Group S. This indicates that the combined intraoperative use of small doses of dobutamine can enhance cardiac systolic function, reduce end-systolic ventricular volume, and increase both the antegrade cardiac blood flow and the pressure gradient between the vena cava and the right atrium during diastole. Consequently, this combined intraoperative use increases hepatic venous blood return and reduces hepatic venous pressure, but also reduces reliance on nitroglycerin for low central venous pressure techniques. In addition, clear operative field was a prominent factor in reducing bleeding in Group D compared to Group S, suggesting that the combined intraoperative use of low-dose dobutamine could reduce intraoperative bleeding and ensure hemodynamic stability.

To improve patient prognosis, it is essential to maintain low central venous pressure, restore the effective circulating blood volume, improve microcirculatory perfusion in tissues and organs, and reduce lactic acid production. LVETc is obtained by esophageal ultrasound via a transgastric fundus long-axis section, and the Doppler method is used to measure the left ventricular ejection time in the left ventricular outflow tract and correct for HR. Dynamic changes in LVETc and SV were monitored by Transesophageal Echocardiography (TEE) to adjust the patient's volume status with high accuracy and satisfactory results^12^. In this study, LVETC was lower than 0.35 s in both groups immediately after induction (T_1_), SBP was decreased, accompanied by a compensatory elevation of HR, indicating that the patients' preoperative water fasting and the vasodilatory effect of the anesthetic drugs used induced an insufficient circulating blood volume. Intraoperatively, LVETc fluctuated between 0.35 and 0.4 s in both groups, suggesting that the optimal blood volume was promptly adjusted to all patients according to the real-time monitoring of LVETc. SV in group D was higher than that in group S at each time point during the operation, indicating that the effective circulating blood volume and cardiac output were greater in group D compared to group S, which could also partly explain the greater urine volume in group D compared to group S.

Lactate, DO_2_I, and O_2_ERe are physiological indicators that reflect the quality of tissue perfusion. The present study showed that during hepatic surgery portal block, the concentration of anaerobic metabolites, such as adenosine and lactate, increases due to their entering circulation after the hepatic portal is opened, while the liver and kidneys fail to clear these metabolites on time. However, lactate was still within the normal range, and the tissue perfusion status was not significantly affected, in accordance with the report published by Fawcett et al.^13^. Nonetheless, there were no significant changes in DO_2_I and O_2_ERe in both groups at the end of surgery compared with the values registered before induction, suggesting that intraoperative fluid management in both groups could improve tissue microcirculatory perfusion and ensure the balance of oxygen supply and demand.

Although both the abdominal pneumoperitoneum pressure and the negative pressure siphoning phenomenon generated by low central venous pressure allow CO_2_ to enter the venous system and return to the right heart, the benefits derived from the CLCVP technique are more clinically significant than those derived from the lower incidence of air emboli^14^. In this paper, TEE was used, and despite its presence, CO_2_ could be quickly dissolved in the blood without causing any fatal damage to the body. Moreover, the use of dobutamine in group D patients led to a decrease in the use of nitroglycerin, a relative decrease in hepatic venous pressure, a decrease in the pressure difference between the two sides of the broken vein, and a smaller amount of air bubbles entering through the broken vein. In addition, the intraoperative laparoscopic pneumoperitoneum pressure in this study was ≤ 12 mmHg relatively safe and the incidence of air embolism was low, and these findings are consistent with the results of the MakabeK et al*.* study^15^.

In conclusion, our study demonstrated an effective method in enhancing myocardial contractility by dobutamine, reducing reliance on nitroglycerin for low central venous pressure techniques, and reducing the incidence of hypotension and air embolism. Meanwhile, LVETc detection by esophageal ultrasound could efficiently guide volume management in patients undergoing partial hepatectomy, ensuring hemodynamic stability and improving the balance of oxygen supply and demand in tissue microcirculation.

## Data Availability

The datasets analyzed during the current study are available from the corresponding author upon reasonable request.

## Abbreviations

LVET: Left ventricular ejection time
SV: Stroke volume
CVP: Central venous pressure
MAP: Mean arterial pressure
HR: Heart rate
SV: Stroke volume
CLCVP: Controlled low central venous pressure
SBP: Systolic blood pressure
SpO2: Pulse oximetry
ECG: Electrocardiogram
PEtCO2: End-tidal carbon dioxide tension
BIS: Bispectral index
TEE: Transesophageal echocardiography
